# Utilization of dental services among low and middle income pregnant, post-partum and six-month post-partum women

**DOI:** 10.1186/s12903-020-01076-9

**Published:** 2020-04-20

**Authors:** Jagan Kumar Baskaradoss, Amrita Geevarghese

**Affiliations:** 1 Division of Dental Public Health, Department of Developmental and Preventive Sciences, Faculty of Dentistry, Kuwait University, 13110 Kuwait City, Kuwait; 2 Resident, Dental Public Health, Department of Preventive and Restorative Dental Sciences, School of Dentistry, University of California San Francisco, 707 Parnassus Avenue, San Francisco, CA, 94143 USA

## Abstract

**Background:**

This study aims to explore the difference in the utilization pattern of dental services among pregnant, post-partum and six-month post-partum women.

**Methods:**

This cross-sectional questionnaire survey was performed at two maternity and child care hospitals in India that primarily cater to middle and low income communities. Data were collected from 3 groups: 1) pregnant women in their first trimester; 2) post-partum women (< 48 h after delivery); and 3) six-month post-partum women. The primary outcome of interest was dental service utilization during pregnancy. Self-perceived oral health (SPOH) was calculated based on the four global dimensions- knowledge, function, quality of life and social. Multiple logistic regression analysis was carried out to assess the effect of each independent variable after adjustment for the effect of all other variables in the model.

**Results:**

Responses of 450 (150 pregnant, 150 post-partum and 150 six-month post-partum) women were analyzed (response rate = 72%). Significant differences in the dental attendance pattern was observed between the study groups (*p* < 0.01). Dental attendance among pregnant and six-month post-partum women were 60 and 75%, respectively, however, only about 15% of the post-partum women reported to have sought dental care within the 6 months prior to the study. Post-partum women had the highest SPOH scores, indicating poor self-perceived oral health, followed by pregnant and then six-month post-partum women, which was statistically significant (*p <* 0.05). A significantly higher percentage of post-partum women reported to have poor oral and general health, as compared to both, pregnant and six-month post-partum women (*p <* 0.01). Higher percentage of women reporting ‘good’ oral and general health had sought dental care compared with others (*p <* 0.01). After adjusting for all the other variables in the model, women with lower levels of education (OR^a^ = 1.42; 95% CI: 1.01–2.00), women with poor self-perceived oral health (OR^a^ = 1.08; 95% CI: 1.02–1.14) and post-partum women (OR^a^ = 0.15; 95% CI: 0.09–0.24) were found to be less likely to seek regular dental care.

**Conclusion:**

Pattern of dental service utilization among women in this population varied according to their pregnancy status, level of education and self-perceived oral health.

## Background

Pregnancy is associated with a number of psychological and hormonal changes in women [1]. An increase in the secretion of hormones like estrogen and progesterone leads to gingival inflammation [2]. Maintaining good oral health before, during and after pregnancy is important not only for the mother but also for the health of the infant. Studies have shown that maternal periodontitis can lead to preterm low birth weight babies [3–5]. Poor maternal oral health during pregnancy has been associated with early childhood caries and also long term systemic disorders for the newborn [6]. From a psychological perspective, poor periodontal health is associated with poor oral health related quality of life among pregnant women [7].

The oral health of pregnant women is influenced by various socio-cultural factors including education and socio-economic status [8]. Limited access to oral health resources, medical conditions that influence oral health, and limited knowledge of the relationship between oral and general health might be the reason for this association [9].

**Fig. 1:**
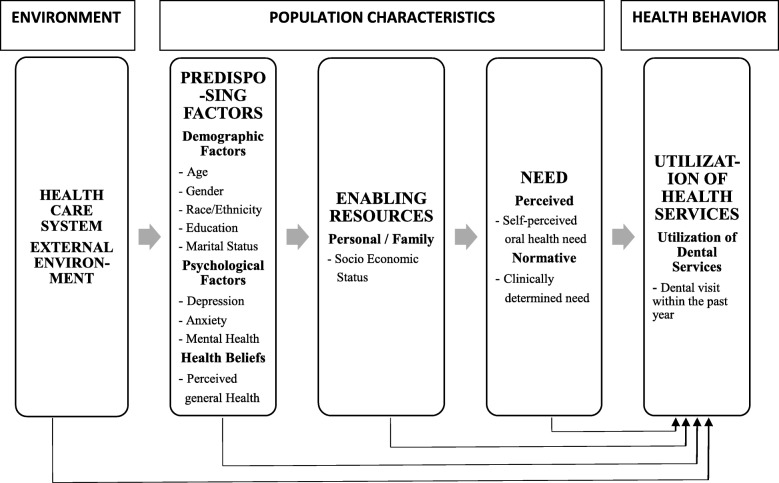
Andersen’s emerging behavioral model of health services use

The dental need could either be ‘perceived need’, which takes into account the individuals’ self-perception of their oral health or ‘normative need’, which is the professionally determined need, based on the subjects’ clinical findings [10]. Relying solely on normative methods without integrating the individual’s perception of dental health may lead to overestimation of dental care needs [11]. Along with the concept of need, several other factors like patient’s socio-demographic characteristics and availability of dentists also influence the dental service utilization. Andersen’s [12] behavioral model of health services use has been widely used in dentistry to explain the predictors of dental service utilization (Fig. 1).

Most dental procedures can be performed safely in pregnant women [13]. Several organizations have put forth the recommendation that pregnant women should undergo routine dental prophylaxis during pregnancy. The American Academy of Pediatric Dentistry recommends that all women should receive a comprehensive oral health evaluation and risk assessment during pregnancy [14]. The American Academy of Periodontology also recommends that pregnant women with periodontitis receive treatment during pregnancy [15]. The American College of Obstetricians and Gynecologists recommend that women should routinely be counseled about the maintenance of good oral health habits throughout their lives as well as the safety and importance of oral health care during pregnancy [16]. However, pregnant women often defer necessary dental treatment until after delivery [15]. Several factors have been reported in the literature that deter women from seeking dental care during pregnancy, such as socio-economic and socio-cultural factors, lack of public awareness of the importance of oral health and concerns for fetal safety during dental treatment [17].

Several studies have reported on the oral health practices among pregnant women [18, 19]. However, minimal information is available on the utilization of dental services among low and middle income women. The aim of this study was to explore the difference in the utilization pattern of dental services among low and middle income pregnant, post-partum and six-month post-partum women.

## Methods

This cross-sectional questionnaire survey was performed at two of the largest maternal and child care hospitals (Women and Child Hospital and Sri Avittom Tirunal Hospital) in the State of Kerala, India. Written consent was obtained from the concerned authorities of the two hospitals. This study was conducted in accordance with the World Medical Association Declaration of Helsinki and approved by the Institutional Ethics Committee of Sree Chitra Tirunal Institute of Medical Science and Technology, Kerala, India. This study is reported based on the guidelines of strengthening the reporting of observational studies in epidemiology (STROBE) [20].

Recruitment of study participants were carried out at three different sites at these hospitals: 1) pregnant women in their first trimester-when they came for their routine antenatal consultation at the outpatient clinic of Obstetrics and Gynecology department; 2) post-partum women – after they were shifted to the post-partum labor wards (within 48 h’ after delivery) and 3) six-month post-partum women- when they bring their child for the sixth-month immunization at the immunization clinics of the respective hospitals. The sample size calculation was based on an estimation of the proportion of women utilizing dental services during pregnancy in India. Assuming 50% of women utilizing dental services [18] and assuming a difference of 20% in the utilization rates between pregnant and non-pregnant women, it was calculated that 103 subjects per group were required for achieving a power of 80% and a two-sided confidence interval (CI) of 95%. This was rounded to 150 per group.

Only women between the age group of 18–35 years were invited to participate in this study. Women presenting with medically compromised conditions or those who reported to be under antibiotic therapy were excluded from the study. The investigators referred to the hospital registers to select the subjects. A structured questionnaire was administered in two parts: the first section on dental information and the second section on the utilization pattern of dental services. Sociodemographic data including age, education, religion, period of gestation, employment and socio economic status (SES) were also collected. In the study, SES was calculated based on the standard of living index (SLI) with minor modifications. This index is similar to the one used in National Family Health Survey (NFHS) II [21]. SLI was calculated on the basis of income, possession of goods, toilet facilities and the type of cooking fuel used by the respondents. Based on this score SES was divided into ‘high’, ‘medium’ and ‘low’ categories. The frequency of eating sugary foods was also calculated from the sum of occasions on which cakes, biscuits, confectionery, table sugar, jams and soft drinks were consumed in the previous 24 h. The total score was then categorized as low (< 3 times/day), medium (3–5 times/day) and high (> 5 times/day). The dental beliefs of women were explored using 2 focused questions: 1) ‘Do you believe that dental diseases could lead to other medical conditions?’ and 2) ‘Do you believe that bleeding from the gums is normal during pregnancy?’. Self-perceived oral health (SPOH) was calculated based on the four global dimensions that was adopted from the study by Ekback et al. [22] Higher SPOH scores indicate poorer self-perceived oral health status. In this study, knowledge was assessed by three questions, function by three questions, quality of life by one question and social by two questions. Overall oral health and general health indicator was assessed by two self-rating questions ‘How would you rate your oral/general health?’ respectively, with the response categories: 1) Good, 2) Fair, 3) Poor. The questionnaire was pilot tested in a small group of 10 women conveniently chosen from the selected hospitals.

The primary outcome of interest was participants’ dental service utilization, dichotomized as whether or not a woman reported having had a routine dental visit in the previous 6 months. Routine dental visit as a dental checkup or dental prophylaxis. Emergency dental visits are not included in this definition. The data from completed questionnaires were analyzed and the descriptive frequency tables were generated. Cross-tabulation with the chi-squared test was used to evaluate the differences between the different variables. Difference in the SPOH scores between the groups was analyzed using Analysis of variance (ANOVA) with Tukey’s post-hoc multiple comparisons. Multiple logistic regression analysis was carried out to assess the effect of each independent variable after adjustment for the effect of all other variables in the model. *P*-value of < 0.05 was considered significant. All statistical analyses were carried out using SPSS 17.0 software (Statistical Package for the Social Sciences for Windows®; SPSS Inc., Chicago, IL, USA).

## Results

A total of 450 women participated in the study (response rate = 72%). The response rate was similar across the groups. Table 1 shows the participants’ socio-demographic and dental characteristics stratified by pregnancy status and dental care utilization. About 40% of the respondents were from Urban areas and about 47% had completed secondary school education. Most of the participants were unemployed (80.4%) and belonged to a low income category (70.2%). About 50% of the respondents had sought regular dental care within the previous 6 months.

**Table 1:** Socio-demographic and dental characteristics of the study groups stratified by their pregnancy status and utilization of dental services

Variables		Pregnancy Status				Sought Dental Care (last 6 months)	
	Total	Six-month post-partum women	Pregnant women	Post-Partum women	***P*** value*	Yes	***P*** value*
	450 (100.0) N (%)	150 (33.3) N (%)	150 (33.3) N (%)	150 (33.3) N (%)		224 (49.8) N (%)	
**Place of Residence**							
Urban	143 (39.6)	31 (37.3)	49 (38.3)	63 (42.0)	0.73	61 (42.6)	0.28
Rural	218 (60.4)	52 (62.7)	79 (61.7)	87 (58.0)		106 (48.6)	
**Religion**							
Hindu	303 (67.3)	98 (65.3)	89 (59.3)	116 (77.3)	0.02	140 (46.2)	0.04
Christian	86 (19.1)	31 (20.7)	37 (24.7)	18 (12.0)		53 (61.6)	
Muslim	61 (13.6)	21 (14.0)	24 (16.0)	16 (10.7)		31 (50.8)	
**Education**							
Primary and Secondary	212 (47.1)	45 (30.0)	68 (45.3)	99 (66.0)	< 0.01	84 (39.6)	< 0.01
Higher Secondary	146 (32.4)	68 (45.3.)	40 (26.7)	38 (25.3)		83 (56.8)	
Graduate and Above	92 (20.4)	37 (24.7)	42 (28.0)	13 (8.7)		57 (61.9)	
**Occupation**							
Unemployed	362 (80.4)	96 (64.0)	127 (84.7)	139 (92.7)	< 0.01	167 (46.1)	< 0.01
Employed	88 (19.6)	54 (36.0)	23 (15.3)	11 (7.3)		57 (64.7)	
**Socio-economic status**							
Middle	134 (29.8)	39 (26)	46 (30.7)	49 (32.7)	0.41	60 (44.8)	0.18
Low	316 (70.2)	111 (74.0)	104 (69.3)	101 (67.3)		164 (51.9)	
**Frequency of eating sugary foods**							
< 3 times/day	149 (33.1)	64 (42.7)	34 (22.7)	51 (34.0)	< 0.01	77 (51.7)	0.85
3–5 times/day	211 (46.9)	60 (40.0)	77 (51.3)	74 (49.3)		103 (48.8)	
> 5 times/day	90 (20.0)	26 (17.3)	39 (26.0)	25 (16.7)		44 (48.9)	
**Frequency of brushing**							
Regular (Brush > 1/day)	240 (53.3)	87 (58.0)	85 (56.7)	68 (45.3)	0.09	126 (52.5)	0.30
Irregular (Brush ≤1/day)	210 (46.7)	63 (42.0)	65 (43.3)	82 (54.7)		98 (46.7)	
**Bleeding while brushing**							
No	230 (51.1)	99 (66.0)	51 (34.0)	80 (53.3)	< 0.01	117 (50.9)	0.64
Yes	220 (48.9)	51 (34.0)	99 (66.0)	70 (46.7)		107 (48.6)	
**Dental problems (last 6 months)**							
No	211 (46.9)	82 (54.7)	58 (38.7)	71 (47.3)	0.02	95 (45.0)	0.06
Yes	239 (53.1)	68 (45.3)	92 (61.3)	79 (52.7)		129 (54.0)	
**Sought dental care (last 6 months)**							
No	226 (50.2)	38 (25.3)	60 (40.0)	128 (85.3)	< 0.01		
Yes	224 (49.8)	112 (74.7)	90 (60.0)	22 (14.7)			
**Barriers to dental care during pregnancy**							
Lack of need	111 (24.7)	63 (42.0)	30 (20.0)	18 (12.0)	< 0.01	68 (61.3)	< 0.01
Fear	38 (8.4)	11 (7.3)	13 (8.7)	14 (9.3)		15 (39.5)	
Concern for child’s safety	249 (55.3)	61 (40.7)	94 (62.7)	94 (62.7)		124 (49.8)	
Financial issues	52 (11.6)	15 (10.0)	13 (8.7)	24 (16.0)		17 (32.7)	
**Advised on seeking dental care**							
No	402 (89.3)	133 (88.7)	136 (90.7)	133 (88.7)	0.81	199 (49.5)	0.43
Yes	48 (10.7)	17 (11.3)	14 (9.3)	17 (11.3)		25 (52.1)	
**Dental diseases lead to other medical conditions**							
No	419 (93.1)	133 (88.7)	136 (90.7)	150 (100.0)	< 0.01	203 (48.4)	0.04
Yes	31 (6.9)	17 (11.3)	14 (9.3)	0 (0.0)		21 (67.7)	
**Bleeding from gums is normal during pregnancy**							
No	124 (27.6)	41 (27.3)	43 (28.7)	40 (26.7)	0.99	61 (49.2)	0.48
Yes	326 (72.4)	109 (72.7)	107 (71.3)	110 (73.3)		163 (52.0)	
**Self-reported oral health status**							
Good	43 (9.6)	26 (17.3)	8 (5.3)	9 (6.0)	< 0.01	26 (60.5)	< 0.01
Moderate	284 (63.1)	108 (72.0)	96 (64.0)	80 (53.3)		151 (53.2)	
Poor	123 (27.3)	16 (10.7)	46 (30.7)	61 (40.7)		47 (38.2)	
**Self-rating of general health**							
Good	58 (12.9)	46 (30.7)	9 (6.0)	3 (2.0)	< 0.01	39 (67.2)	< 0.01
Moderate	268 (59.6)	92 (61.3)	98 (65.3)	78 (52.0)		135 (50.4)	
Poor	124 (27.6)	12 (8.0)	43 (28.7)	69 (46.0)		50 (40.3)	

*Chi-squared test

Religion, education and occupation were significantly different in terms of pregnancy status and dental care utilization. Lower percentage of post-partum women (8.7%) had graduate or higher level education as compared with pregnant (28.0%) or six-month post-partum (24.7%) women, which was statistically significant (*p* < 0.01). An increasing trend in the utilization of dental services was observed with an increase in the level of education of the participants (*p* < 0.01). Higher percentage of six-month post-partum women were employed (36.0%) as compared with pregnant (15.3%) or post-partum (7.3%) women (*p* < 0.01). Diet was not significantly associated with dental service utilization, though pregnant women reported to have a higher frequency of consumption of sugary foods (*p* < 0.01). More pregnant women (66.0%) reported to have bleeding while brushing as compared with post-partum women (46.7%) or six-month post-partum (34.0%) women, which was statistically significant (*p* < 0.01). Similarly, higher percentage of pregnant women (61.3%) reported experiencing dental problems in the last 6 months as compared with post-partum (52.7%) or six-month post-partum (45.3%) women (*p =* 0.02). Dental service utilization was significantly higher among women who reported experiencing dental problems (*p =* 0.06). A significant difference in the dental attendance pattern was observed between the study groups (*p* < 0.01). Dental attendance among six-month post-partum women and pregnant were about 75 and 60%, respectively, however, only about 15% of the post-partum women reported to have sought dental care in the 6 months prior to the study. The main barrier to dental care during pregnancy was reported to be ‘concern for the safety of the child’ followed by ‘financial issues’ and ‘fear of the dentist’. This was again significantly different between pregnant, post-partum and six-month post-partum women, with a higher percentage of pregnant and post-partum women citing ‘concern for the child’s safety’ as the primary barrier to seeking care (*p <* 0.01). Majority of the respondents did not report receiving any advice on the need to seek oral health care during pregnancy. Only about 7 % of the respondents believed that oral disease could lead to other medical conditions. This was significantly different across the groups and also in terms of dental care utilization. About three-fourth of the respondents believed that bleeding from the gums was normal during pregnancy. This was not significantly different between the groups.

A significantly higher percentage of post-partum women reported to have poor oral and general health (40.7 and 46.0% respectively), as compared to both, pregnant and six-month post-partum women (*p <* 0.01). Higher percentage of women reporting ‘good’ oral and general health had sought dental care compared with others (*p <* 0.01).

Table 2 shows the distribution of the responses to the 4 global dimensions (Knowledge, Function, Quality of life and Social) of self-perceived oral health by pregnant status. The self-rating for all the dimensions were significantly poorer for the post-partum women as compared with pregnant or six-month post-partum women. Total SPOH scores was significantly different between the three groups (Table 3). Post-partum women had the highest SPOH scores, indicating poor self-perceived oral health, followed by pregnant and then six-month post-partum women, which was statistically significant (*p <* 0.05).

**Table 2:** The 9 questions covering the four global dimensions of self-perceived oral health

**Knowledge**						
	**Expert level of Knowledge**	**Good Knowledge**	**Fair Knowledge**	**Some Knowledge**	**No Knowledge**	*P*value*
Q 1. Do you know the mechanism behind caries and periodontal diseases?						
Six-month post-partum women	11 (7.3)	30 (20.0)	9 (6.0)	32 (21.3)	68 (45.3)	< 0.01
Pregnant	1 (0.7)	5 (3.3)	28 (18.7)	54 (36.0)	62 (41.3)	
Post-Partum	3 (2.0)	18 (12.0)	26 (17.3)	55 (36.7)	48 (32.0)	
Q 2. Do you know how to avoid caries and periodontal diseases?						
Six-month post-partum women	7 (4.7)	29 (19.3)	8 (5.3)	37 (24.7)	69 (46.0)	< 0.01
Pregnant	0 (0.0)	2 (1.3)	21 (14.0)	68 (45.3)	59 (39.3)	
Post-Partum	2 (1.3)	18 (12.0)	44 (29.3)	57 (38.0)	29 (19.3)	
Q 3. Do you know that it is important to use fluoridated toothpaste?						
Six-month post-partum women	12 (8.0)	57 (38.0)	39 (26.0)	40 (26.7)	2 (1.3)	< 0.01
Pregnant	10 (6.7)	23 (15.3)	48 (32.0)	47 (31.3)	22 (14.7)	
Post-Partum	15 (10.0)	38 (25.3)	39 (26.0)	30 (20.0)	28 (18.7)	
**Function**						
	**Never**	**Hardly Ever**	**Occasionally**	**Fairly Often**	**Very Often**	
Q 4. You feel severe pain while drinking hot or cold drinks						
Six-month post-partum women	14 (9.3)	31 (20.7)	78 (52.0)	22 (14.7)	5 (3.3)	< 0.01
Pregnant	1 (0.7)	6 (4.0)	35 (23.3)	59 (39.3)	49 (32.7)	
Post-Partum	1 (0.7)	1 (0.7)	23 (15.3)	70 (46.7)	55 (36.7)	
Q 5. You have headaches due to your teeth or your mouth						
Six-month post-partum women	38 (25.3)	57 (38.0)	44 (29.3)	10 (6.7)	1 (0.7)	< 0.01
Pregnant	14 (9.3)	34 (22.7)	57 (38.0)	29 (19.3)	16 (10.7)	
Post-Partum	3 (2.0)	20 (13.3)	40 (26.7)	56 (37.3)	31 (20.7)	
Q 6. You have difficulty eating food						
Six-month post-partum women	57 (38.0)	56 (37.3)	29 (19.3)	8 (5.3)	0 (0.0)	< 0.01
Pregnant	18 (12.0)	34 (22.7)	42 (28.0)	33 (22.0)	23 (15.3)	
Post-Partum	11 (7.3)	17 (11.3)	33 (22.0)	49 (32.7)	40 (26.7)	
**Quality of Life**						
Q 7. You feel depressed due to your teeth or your mouth						
Six-month post-partum women	51 (34.0)	61 (40.7)	32 (21.3)	6 (4.0)	0 (0.0)	< 0.01
Pregnant	12 (8.0)	52 (34.7)	55 (36.7)	29 (19.3)	2 (1.3)	
Post-Partum	6 (4.0)	38 (25.3)	32 (21.3)	46 (30.7)	28 (18.7)	
**Social**						
Q 8. You avoid laughing due to your teeth or your mouth						
Six-month post-partum women	37 (24.7)	47 (31.3)	50 (33.3)	15 (10.0)	1 (0.7)	< 0.01
Pregnant	3 (2.0)	25 (16.7)	45 (30.0)	43 (28.7)	34 (22.7)	
Post-Partum	1 (0.7)	12 (8.0)	40 (26.7)	58 (38.7)	39 (26.0)	
Q 9. You feel embarrassed due to your teeth or your mouth						
Six-month post-partum women	53 (35.3)	53 (35.3)	34 (22.7)	10 (6.7)	0 (0.0)	< 0.01
Pregnant	7 (4.7)	41 (27.3)	45 (30.0)	32 (21.3)	25 (16.7)	
Post-Partum	5 (3.3)	21 (14.0)	33 (22.0)	61 (40.7)	30 (20.0)	

*Chi-squared test

**Table 3:** Total self-perceived oral health score

Total SPOH scores	Mean	SD	***P*** Value*
Six-month post-partum women	12.43	4.29	< 0.05†§
Pregnant	22.16	5.05	< 0.05§¥
Post-Partum	23.74	3.37	< 0.05†¥

*Analysis of variance (ANOVA) with Tukey’s post-hoc multiple comparisons

† As compared with pregnant women; § As compared with post-partum women; ¥ As compared with six-month post-partum women

The results of the multivariate logistic regression model to assess the significance of variables as predictors of dental attendance (dental visit in the last 6 months – yes/no) are shown in Table 4. Socio-demographic variables were entered in the first step followed by the dental characteristics. In the final step, pregnancy status and SPOH scores were entered into the model. After adjusting for all the other variables in the model, women with lower levels of education (OR^a^ = 1.42; 95% CI: 1.01–2.00), women with poor self-perceived oral health (OR^a^ = 1.08; 95% CI: 1.02–1.14) and post-partum women (OR^a^ = 0.15; 95% CI: 0.09–0.24) were found to be less likely to seek regular dental care.

**Table 4:** Summary of Multivariate logistic regression analysis with dental visit within previous 6 months (yes/no) as the dependent variable

Independent variables	B	SE	OR	95% Confidence Interval
Place of residence	0.29	0.27	1.34	0.78–2.28
Religion	0.20	0.18	1.22	0.86–1.74
Occupation	0.41	0.36	1.5	0.74–3.06
Education	0.35	0.18	1.42	1.01–2.00
Socio-Economic Status	0.51	0.28	1.66	0.96–2.90
Pregnancy Status	−1.92	0.24	0.15	0.09–0.24
SPOH score	0.07	0.03	1.08	1.02–1.14

Nagelkerke R Square = 40.7%

## Discussion

This study was carried out in an urban setting at two maternal and child care hospitals in the State of Kerala, India. The selection of these two hospitals were based on the availability of a large number of accessible pregnant, post-partum and six-month post-partum women. These hospitals cater mostly to the low and middle income women and the cost of treatments are subsidized. This study presents the difference in the dental service utilization pattern among low and middle income women at three distinct phases of pregnancy - first trimester, post-partum and six-month post-partum. Post-partum women were least likely to seek routine dental care compared to pregnant or six-month post-partum women.

Pregnancy is associated with higher incidence of oral conditions like gingivitis and periodontitis [4]. The probable reasons for an increase in the risk for dental diseases during pregnancy might be due to inadequate oral hygiene, medical co-morbidities, limited awareness about the importance of maintaining proper oral hygiene and failure to seek regular dental care [5, 6]. Also, maternal oral health is one of the greatest predictors of childhood oral health [23]. Maternal behaviors, including attention to oral hygiene and dietary practice, may also influence this risk.

Pregnant women generally tend to postpone their dental visits until after delivery due to concern over their child’s health. In the US, less than 50% of pregnant women seek dental care during pregnancy [24, 25]. Similarly, other countries such as Australia (36%), Greece (27%) and UK (33%), have reported poor utilization of dental services by pregnant women [26]. Poor utilization of dental services among pregnant women is well documented worldwide and is evident in India as well. In this study, more than half of the respondents reported having experienced some dental problem in the previous 6 months. However, only less than 15% of the post-partum women sought dental care during the same time period. About three-quarter of the six-month post-partum women and more than half of the pregnant women in the first trimester reported seeking dental care within the 6 months prior to the study. This indicates that most women in this study preferred to seek dental care either before the first trimester or postpone it until after delivery. Concern for child’s safety is a commonly cited barrier for pregnant women seeking dental care even though it is well established that dental treatment during pregnancy is extremely safe and will not result in adverse pregnancy outcomes [17]. In addition, majority of the participants believe that poor oral health is normal during pregnancy.

Only a tenth of the participants recalled being advised on seeking regular dental care during pregnancy. Oral health is often ignored and not considered as a part of regular antenatal care and this finding is strongly supported by several other studies [17, 27]. Lack of awareness among pregnant women about the importance of maternal oral health is associated with poor utilization of dental services [26]. Only over 5 % of the surveyed women in this study believed that dental diseases could lead to other diseases. Similar findings were reported by Boggess et al. [9].

Most pregnant women in this population undergo at least one antenatal care visit during pregnancy [21]. Doctors and midwives could play a vital role in educating pregnant women and improving their awareness about oral health care during pregnancy. This could also address several misconceptions pregnant women may have, which in turn would influence their oral health practice and potentially improve the uptake of dental services.

Education was associated with dental attendance, with women who had completed secondary or higher education were more likely to seek routine dental care. Similar results were found in a systematic review of 25 studies [28]. Educated women may have better knowledge and awareness about the importance of oral health and regular dental visits especially during pregnancy. Also, educated women might have better health literacy, therefore they might be able to navigate the healthcare system better [29]. Similarly, more working women sought routine dental care as compared with unemployed women. Cost of dental services is a significant barrier especially among those belonging to the low socio-economic strata and those with minimal household incomes [26].

In the multivariate model, self-perceived oral health scores were associated with dental service utilization. It should be noted that the four self-reported global dimensions measure problems with mouth and teeth in general and were not restricted to the social and psychological consequences resulting from any particular dental pathology. Differences in the self-perceived oral health between the different groups of women reflect not only the variations in oral conditions but also variations in their attitudes towards oral health.

As described by Andersen [12], health services work towards improving the health status of the population, both as perceived by the population and as evaluated by health care professionals. The Andersen model acknowledges the role of several environmental and population characteristics in determining the need for oral health care, which could be perceived or normative. Though several studies have assessed the normative need of pregnant women, only few studies have explored the intricate relationship between perceived need and utilization of dental services. It is important to understand perceived need as it is closely related to the concept of self-efficacy. Self-efficacy assumes that people adopt self-care practices if they perceive that these practices make a difference [30]. While several programs focus on educating and motivating pregnant women on the importance of good oral hygiene, the role of self-perceived oral health need is often overlooked. This study shows that self-perceived oral health is strongly associated with dental utilization. Therefore, raising the awareness towards oral health among pregnant women might lead to behavior changes that could improve dental utilization during pregnancy. Poor oral health during pregnancy can be detrimental to the mothers’ health and also to the well-being of the baby.

This study describes the plausible role of pregnancy status, educational level and self-perceived oral health in the utilization of dental services. Though the study was pragmatic and carried out in a non-research setting, it managed to get an acceptable response rate from the population studied. However, the results of this study should be interpreted with caution. Since this is a cross-sectional study, the direction of the causal relationship cannot be resolved. It is recognized that several pregnancy-related factors could lead women to perceive their oral health more negatively. It is also understood that dental service utilization is a complex interplay of several factors including availability, accessibility, social and cultural belief’s and finance. Further, additional assessments of pain, halitosis or esthetics were not performed in this study, which may influence the overall oral health perception. More robust longitudinal studies are required to explore the different variables contributing to dental service utilization.

## Conclusions

Pattern of dental service utilization among women in this population varied according to their pregnancy status, level of education and self-perceived oral health. Inclusion of an oral health component to the routine antenatal care in this population may improve awareness regarding the importance of oral health among vulnerable pregnant women.

## Data Availability

The datasets analysed in the current study are available from the corresponding author on reasonable request.

## Abbreviations

NFHS: National family health survey
SPOH: Self perceived oral health
SES: Socio-economic status
SLI: Standard of living index
